# The impact of pregnancy on sexual functioning in Polish women

**DOI:** 10.1007/s00404-024-07648-2

**Published:** 2024-07-30

**Authors:** Edyta Szymańska, Rafał Kisielewski, Lidia Kisielewska, Janusz Tomaszewski

**Affiliations:** 1 Department of Gastroenterology, Hepatology, Feeding Disorders and Pediatrics, The Children’s Memorial Health Institute, Warsaw, Poland; 2 Tomaszewski Gynecology and Obstetrics Private Clinic, Bialystok, Poland; 3 Department of Gynecologic Oncology, The Centre of Oncology, Bialystok, Poland

**Keywords:** Pregnancy, Sexual functions, Female sexual dysfunction

## Abstract

**Introduction:**

Sexual life of pregnant women alters during pregnancy due to the physiologic,’ anatomic and hormonal changes in her body. Therefore, the aim of this study was to evaluate female sexual functioning after becoming pregnant.

**Patients and methods:**

A prospective survey study including 148 pregnant women. An anonymous questionnaire including 60 inquiries concerning intimate relationship before and during pregnancy was performed. The following statistical test were used: Chi-square test of independence, Spearman’s rank correlation coefficient and Wilcoxon test. The significance level of *p* = 0.05 was assumed.

**Results:**

Most of the respondents were between 31 and 40 years old (55%). Majority of them were married (86%). During pregnancy, slightly more than half of women had a moderate need for sexual intercourse (51%), a large percentage of them had a low need (32%), a high need for intercourse was declared by 17% of women. The correlation analysis showed a statistically significant relationship between women’s education and the need for sex before pregnancy (*p* = 0.049). Respondents with higher degrees of education more often felt the need for intercourse before pregnancy. No correlation was found between education and the need for intercourse after pregnancy (*p* = 0.107). After becoming pregnant, 51% of women had less need for intercourse, 7% more, and 42% the same as before pregnancy, and these differences were statistically significant (*p* < 0.001). Also, a decreased satisfaction with sexual intercourse was reported during pregnancy (*p* < 0.001). After getting pregnant, the average number of intercourses decreased in majority (71%) of respondents, and differences in the number of intercourses before and during pregnancy were statistically significant (*p* < 0.001).

**Conclusion:**

Pregnancy has significant impact on woman’s sexuality. After becoming pregnant majority of women declare less need for sexual intercourses, decreased number of intercourses with less satisfaction.

## Introduction

Sexuality is a fundamental, biologic function of every human being. The need for sexual activity changes over time, and is influenced by the age, experience, physical and health condition. Sex is also one of the most important domains of the quality of life (QoL) [1]. Female sexual dysfunction (SD) is a common problem, which affects from 20 to 50% of women and its prevalence increases with age [2, 3]. Moreover, women experience processes that do not affect men, such as menstruation, pregnancy and menopause. Pregnancy changes female’s anatomy, physiology, hormonal and emotional state and alters sexual life of a pregnant woman [4]. According to available data, majority of pregnant women declare the deterioration of their sexual life [5]. They usually experience decreased sexual intercourse with less satisfaction that negatively affect their mood and well-being [6].

However, women’s sexual functioning changes with the progression of her pregnancy. During the first trimester the female body must adapt to neurohormonal changes responsible for inducing drowsiness, mood swings, fatigue, nausea and vomiting, breast enlargement and tenderness, increased frequency of urination, abdominal bloating, shortness of breath and low back pain. These symptoms tend to develop abruptly as early as in 5–8 weeks of gestation and occur daily [7]. Such state has a clear negative impact on female’s sexual desire and her QoL during preagnacy. Thus, first trimester is the time of the greatest fluctuations in the frequency of sexual intercourse: from normal pre-pregnancy activity to complete discontinuation of sexual contacts.

In the second trimester of pregnancy sexual intercourse are usually more frequent and of better quality compared to those during the 1st trimester due to reduction in the physical symptoms of pregnancy, which makes woman feel better [8, 10]. Also, vascular changes in the vagina and vulva may lead to even greater satisfaction than before pregnancy [9].

The last third trimester is the time of the lowest libido and lowest frequency of sexual compared with the previous trimesters of pregnancy [6]. The fear of inducing labor or harming the child makes many couples decide to stop sexual activity [10, 11]. Moreover, the changes in woman’s body shape and in her anatomy may make it difficult to perform sexual intercourse.

However, sexual activity in late pregnancy is not associated with an increased risk of neither severe complications such as: low birthweight, premature rupture of membranes or perinatal death, nor preterm birth [12, 13]. Sexual abstinence also has no clear role in prevention of prematurity [14].

As long as it is comfortable, most sexual positions are allowed during pregnancy as well as oral sex [15].

Sexual dysfunctions are more common during pregnancy—Aydin et al. in their study comparing sexual functions of pregnant and non-pregnant women demonstrated that SD was experienced by 91.08% of pregnant women and 67.61% of control subjects [16].

Various parameters, such as trimester, gravidity, parity, and abortion influence sexuality in different ways. Also, physiologic and psychologic changes experienced by a woman during pregnancy period had impact on her sexual life [18]. In addition, data demonstrate that partners with low educational level and women who experienced preconceptional SD have a higher risk of developing/experience SD during pregnancy [17].

Therefore, the aim of this study was to evaluate female sexual functioning after becoming pregnant.

## Patients and methods

An anonymous self-prepared survey containing 60 inquiries concerning intimate relationship before and during pregnancy was performed on pregnant women who were under the care of a private gynecological clinic in Podlasie (East Poland). Participation in the survey was voluntary. The questionnaire was completed and collected after delivery during observation stay at the gynaecologic word.

The first part of the questionnaire included general and demographic information: respondent’s age, area of residence, education, economic status, type and state of relationship.

The second part was related to sexual life before and after pregnancy: frequency of sexual intercourse, their quality, satisfaction, SD, as well as self-perception of one’s attractiveness, appearance or partner’s perception.

The last part concerned general attitude toward sexuality and sexual counseling provided by the gynecologist.

### Statistics

The significance of the relationship between nominal variables was checked using the Chi-square test of independence.

Correlations between two quantitative or ordinal variables were checked using Spearman’s rank correlation coefficient.

The difference in the assessment of sexuality before pregnancy and sexuality during pregnancy was checked using the Wilcoxon test.

In statistical analyses, the significance level of *p* = 0.05 was assumed. The analyses were performed using the SPSS program.

## Results

### General information

The study included 148 women. Most of them were between 31 and 40 years old (55%) and between 25 and 30 years old (39%). The respondents most often completed college—had higher education (61%). Majority of them lived in voivodeship cities (48%), less frequently in poviat (16%) and commune (16%) cities. The respondents most often described their economic status as good (51%) and satisfactory (33%). Less often as low (3%) or very good (13%) (Table 1).

**Table 1:** Basic socio-demographic information about the surveyed women

		Number	%
Age (years old)	18–24	6	4.1
	25–30	57	38.8
	31–40	82	55.1
	41–50	3	2.0
Education	Basic, technical	12	8.1
	Secondary education	18	12.2
	Higher Ist grade (bachelor degree)	25	16.9
	Higher IInd grade (master degree)	90	6.8
	Higher IIIrd grade (PhD)	3	2.0
Place of residence	Countryside	30	20.4
	Communal town	24	16.3
	Country town	23	15.6
	Provincial city	71	47.6
Economic status	Low	4	2.7
	Satisfactory	49	33.1
	Good	76	51.4
	Very good	19	12.8

Most of the surveyed women were married (86%), the rest were in a civil partnership (13%) or did not provide details of the relationship (1%). The duration of the current relationship was most often from 5 to 10 years (39%) or over 10 years (33%), while the duration of sexual intercourse with the current partner was most often from 5 to 10 years (41%) or over 10 years (30%). The respondents most often had from 1 to 3 sexual partners (69%) (Table 2).

**Table 2:** Information about the respondents’ relationship

		Number	%
Type of the relationship	Civil partnership	19	12.8
	Marriage	127	85.8
	Other	2	1.4
Duration of the relationship (years)	< 2	6	4.1
	2–5	35	23.6
	5–10	58	39.2
	> 10	49	33.1
Duration of sexual intercourse with a current partner (years)	< 2	7	4.7
	2–5	35	23.6
	5–10	61	41.2
	> 10	45	30.4
Number of sexual partners	0	7	4.8
	1–3	101	68.5
	4–8	35	24.0
	9–13	3	2.1
	14–19	1	0.7

### Female sexual functioning during pregnancy

**Fig. 1:**
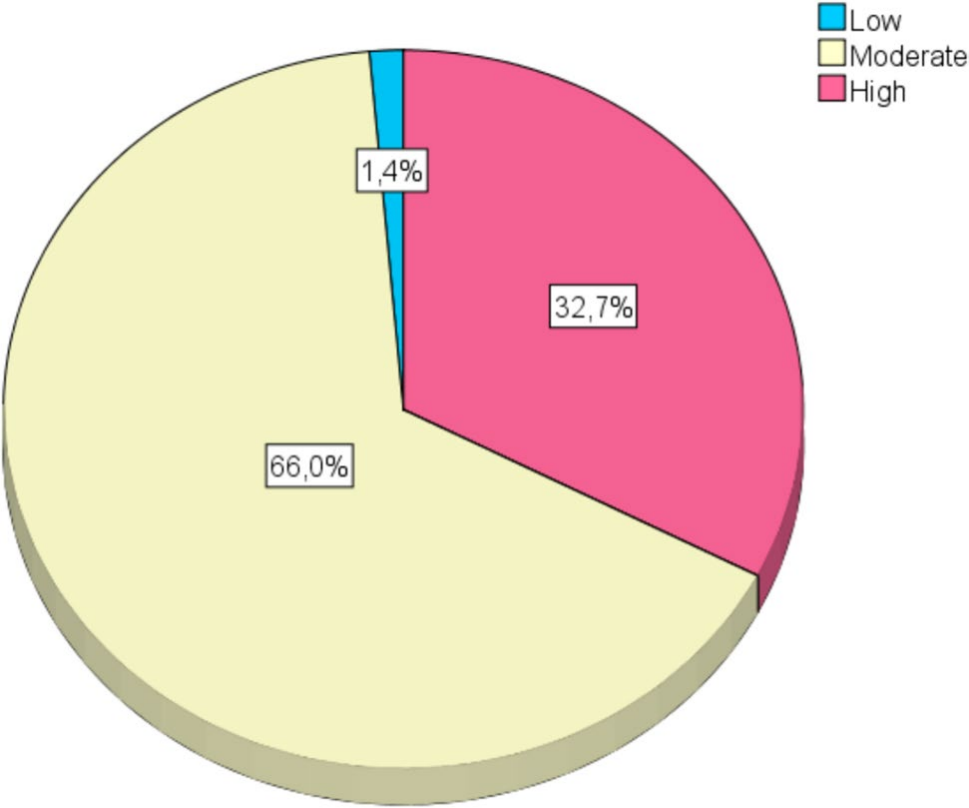
The need for sexual intercourse before pregnancy

Before pregnancy, most women had a moderate need for sex (66%), 33% a high need for sex, a few women had a low need for intercourse (1%) (Fig. 1).

**Fig. 2:**
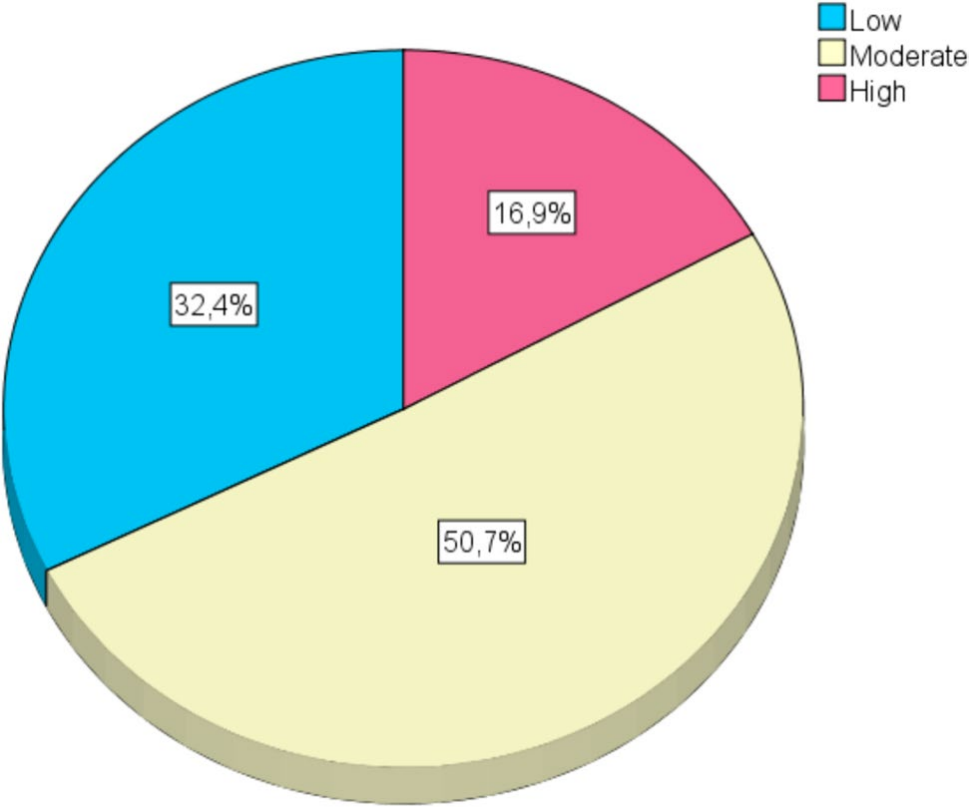
The need for sexual intercourse during pregnancy

During pregnancy, slightly more than half of the women had a moderate need for sex (51%), a large percentage of them had a low need for intercourse (32%), and 17% of women had a high need for intercourse (Fig. 2).

The correlation analysis demonstrated a statistically significant relationship between women’s education and the need for sex before pregnancy (*p* = 0.049, *r* = 0.16). Respondents with higher degrees of education more often felt the need for intercourse before pregnancy. However, no correlation was found between education and the need for intercourse after pregnancy (*p* = 0.107).

After becoming pregnant, 51% of women had less need for intercourse, 7% more, and 42% the same. The analysis using the Wilcoxon test showed that these differences before and during pregnancy were statistically significant (*p* < 0.001).

Respondents were asked to rate their satisfaction with sex using a 5-point scale, where 1 meant very dissatisfied, and 5 meant very satisfied.

The average pre-pregnancy sex satisfaction was 4.4, with a standard deviation of 0.76. The median distribution was 5.0. The rating most often given by female respondents was 5. The average sex satisfaction during pregnancy was 3.6, with a standard deviation of 1.17. The median distribution was 4.0. The rating most often given by the respondents was 3.

After becoming pregnant, 55% of women experienced a decrease in satisfaction with sex, 7% increased satisfaction, and 38% of women did not notice any changes in this matter. The analysis using the Wilcoxon test showed that the discussed differences in the assessment of satisfaction with sex before and during pregnancy were statistically significant (*p* < 0.001). Correlation analysis showed statistically significant relationships between satisfaction with sex before pregnancy (*p* = 0.003) and during pregnancy (*p* = 0.001) and place of residence. Women from smaller towns were more satisfied with sex.

**Fig. 3:**
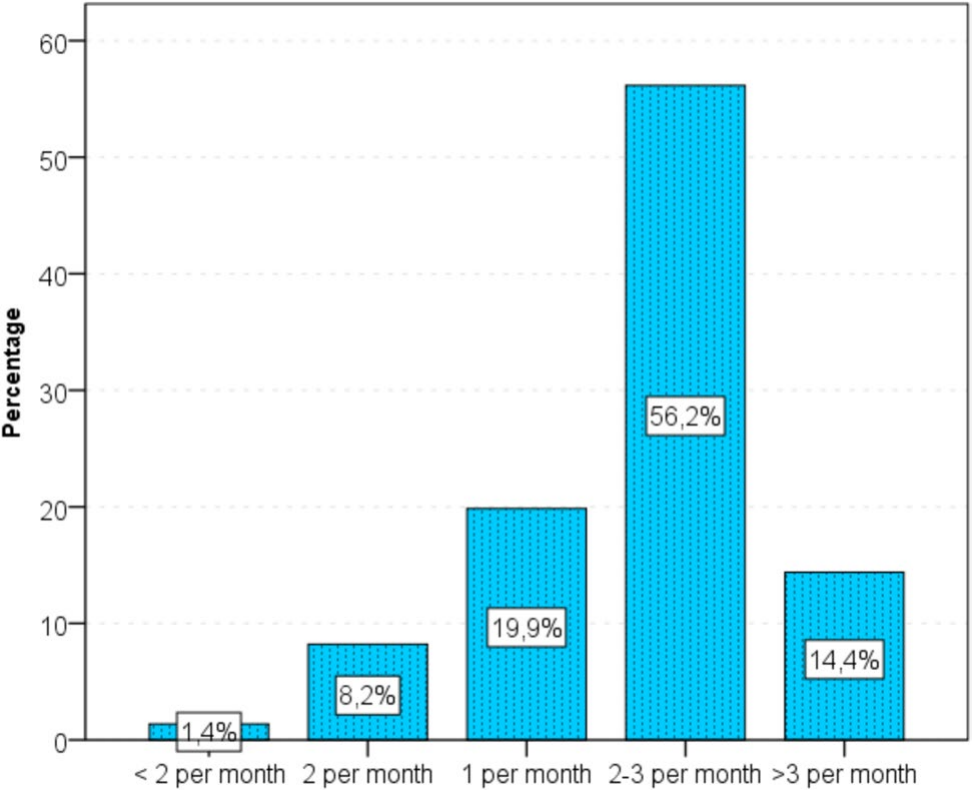
The number of sexual intercourse before pregnancy

**Fig. 4:**
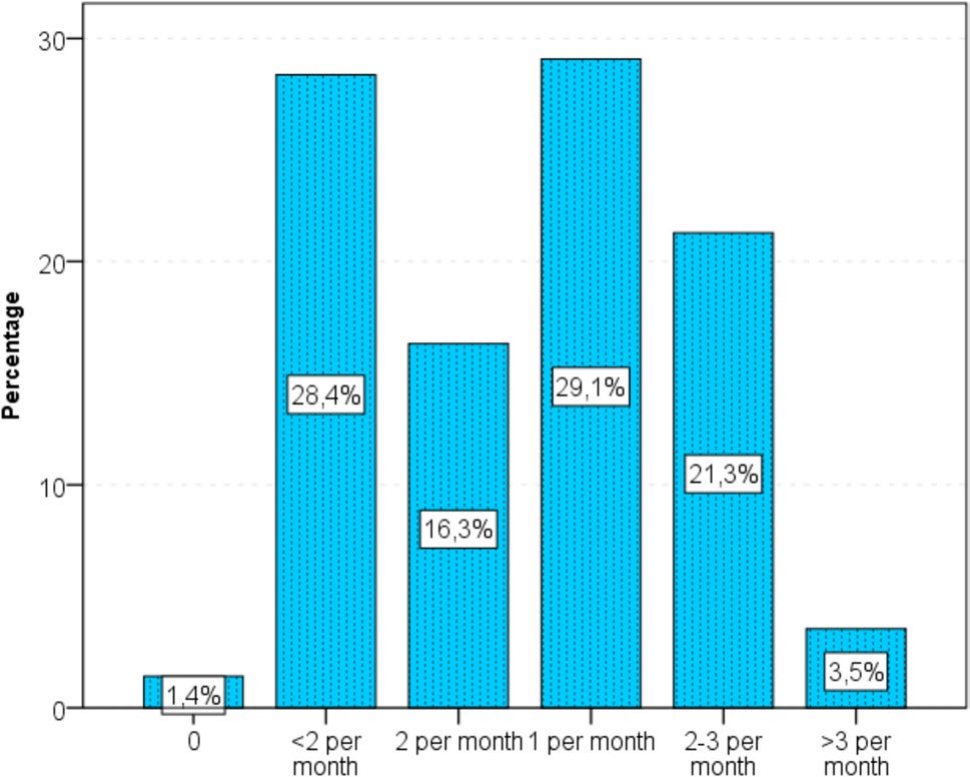
The number of sexual intercourse during pregnancy

After becoming pregnant, the average number of intercourse decreased in 71% of respondents. The analysis using the Wilcoxon test showed that the discussed differences in the number of intercourse, before and during pregnancy were statistically significant (*p* < 0.001). Figure 3 and 4 demonstrate the number of intercourse, before and after pregnancy.

Statistical analysis showed a significant relationship between the average number of intercourses before pregnancy and the length of relationship with the current partner (*p* = 0.015). The correlation was negative and of low strength (*r* = 0.20). Respondents with a longer period of cohabitation with their current partner had fewer intercourses before pregnancy. However, there were no statistically significant relationships between the average number of intercourses during pregnancy and the length of time with the current partner (*p* = 0.526).

After pregnancy, the average duration of intercourse was most often 10 to 15 min (45%), less frequently it was 5 to 9 min (27%) or 20 to 45 min (22%). Very rarely, the mean duration of intercourse was less than 4 min (5%) or longer than 46 min (1%) While, after becoming pregnant, the average duration of intercourse, including foreplay, decreased for 46% of the women, increased for 1%, and more than half of the respondents felt no change (53%). The analysis using the Wilcoxon test showed that the discussed differences in the duration of intercourse before and during pregnancy were statistically significant (*p* < 0.001).

The proffered sex position before pregnancy was the “classic” position (44%), less often the position “me on top” (29%), “doggystyle (21%) or other (9%).

During pregnancy, the preferred sexual position was the "lateral rear to partner” position (42%) and the “classic” position (32%). Less often doggy style (15%) or other (12%).

Slightly more than 32% of the surveyed women had an orgasm before pregnancy, but after becoming pregnant they achieved it less and less often, 57% claimed that pregnancy did not negatively affect the achievement of orgasm, and the remaining respondents did not achieve orgasm both before and during pregnancy (10%).

**Fig. 5:**
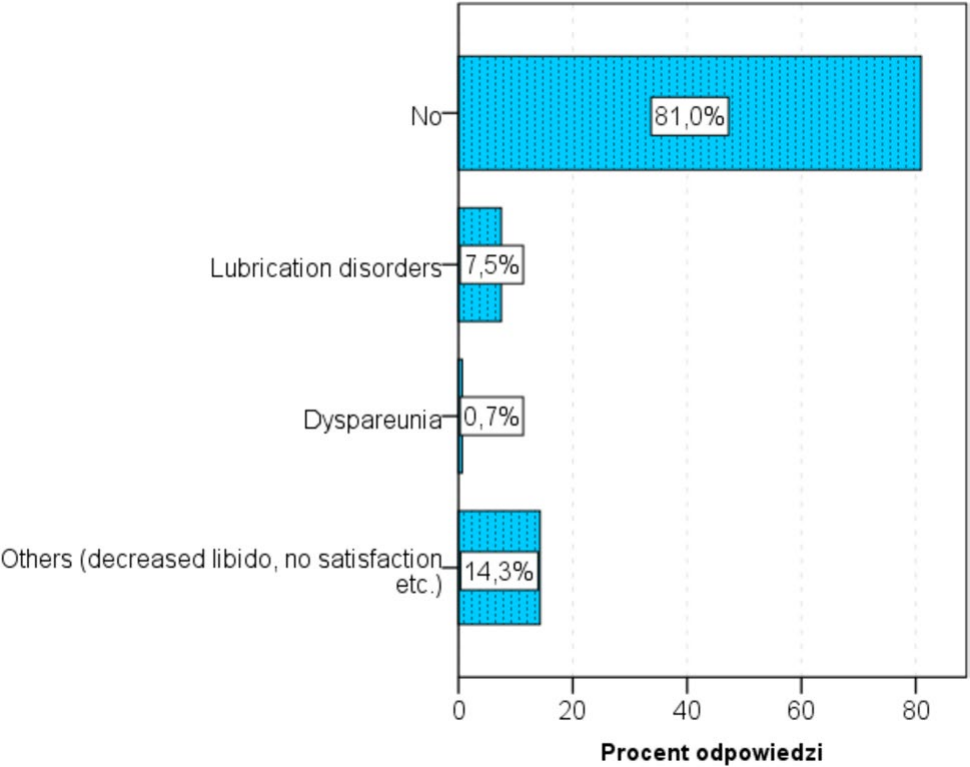
Sexual dysfunctions experienced during pregnancy

Most of the respondents did not experience any SD before becoming pregnant (94%). Few experienced lubrication disorders (3%), pain during intercourse (1%) or other disorders (5%). During pregnancy, 8% of them experienced lubrication disorders, less than 1% experienced pain during intercourse, and 14% had other disorders (Fig. 5).

### General information on the pregnancy and attitude toward sex and its perception after getting pregnant

Nearly 26% of the surveyed women had a history of miscarriages or any other complications related to pregnancy (including abortion).

In most cases, the pregnancy was natural (94%), in 6% of the surveyed women it was in vitro. The vast majority of the surveyed women did not get pregnant using contraception (99%).

Nearly 7% of the surveyed women claimed that their partner currently uses a condom during intercourse during pregnancy.

For the majority of respondents, getting pregnant did not change their attitude toward sex (65%), 12% changed for the better, and for 24% it changed for the worse. More than half of the respondents assessed their attractiveness after getting pregnant worse (57%), 34% the same, and 9% better than before pregnancy.

On the other hand, the majority of the respondents’ partners assessed the attractiveness of the respondent in the same way as before the pregnancy (77%), only 12% assessed it better or worse.

Most of the respondents did not consider that after becoming pregnant they would be less attractive to their partner and that pregnancy would adversely affect their relationships and sexual life (35%), 23% had no such concerns, 23% had them now, and 19% had them before pregnancy but stopped having them during pregnancy.

The majority of the surveyed women did not change their partner’s erotic perception after getting pregnant (57%), 13% of the respondents felt greater desire for their partner, and for 30% the erotic sphere was less important now, and the pregnancy and child were the most important.

**Fig. 6:**
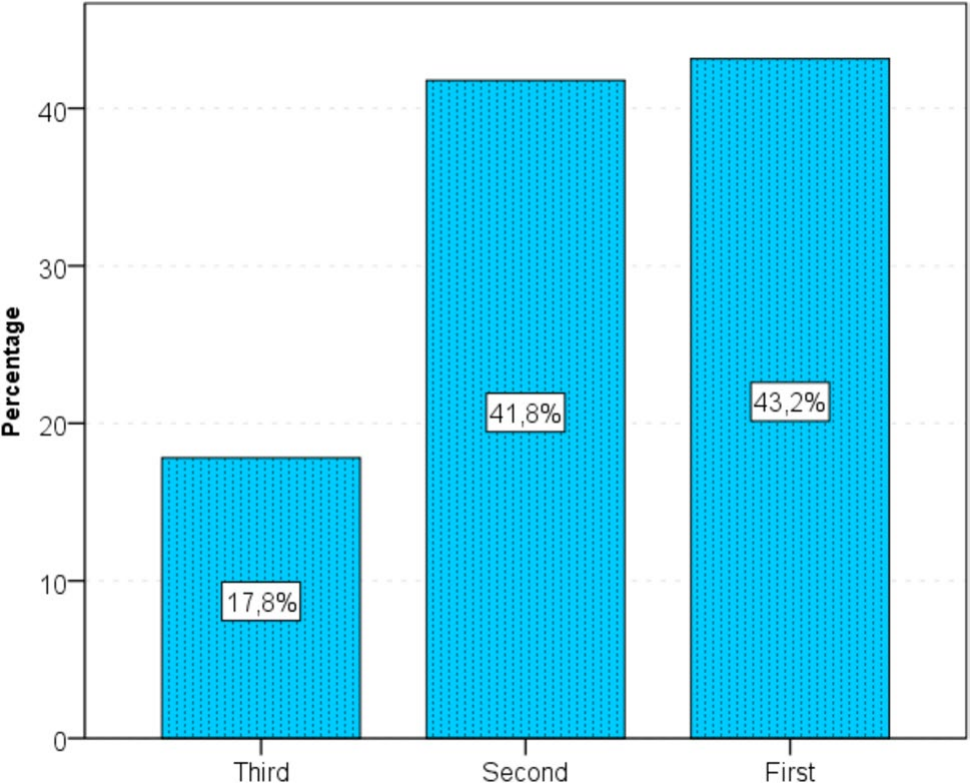
The highest need for sexual intercourse during pregnancy according to certain trimester

Most respondents believed that the lowest sexuality was in the third trimester (66%), less often in the first (28%) or second (6%) trimester, while the highest need for sex was in the third and second trimester–43.2 and 41.8, respectively. (Fig. 6.)

**Fig. 7:**
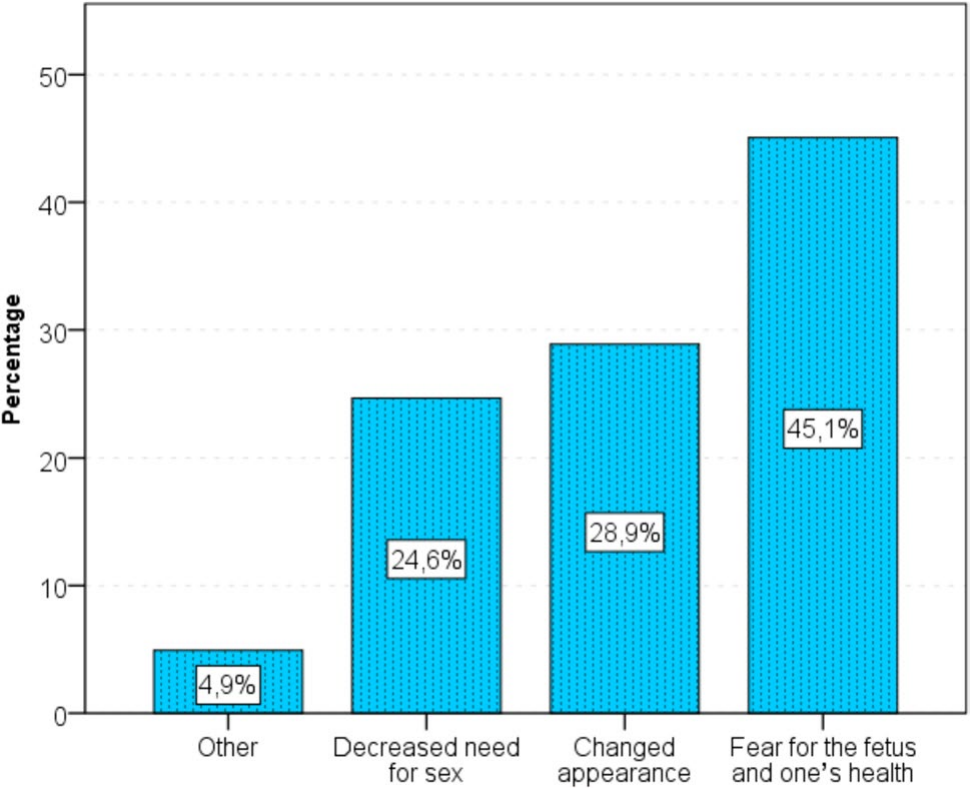
Reasons for restraint from sex during pregnancy

Majority of pregnant women were discouraged from having sexual intercourse during pregnancy due to fear of pregnancy and their health (45%), less frequent physicality (29%), reduced desire for sex (25%) (Fig. 7). The most common reason to limit or give up intercourse was: pregnancy pathology (76%).

### General attitude toward sexuality

For nearly 63% of the surveyed women, sex in a relationship played an important role, 22% of the respondents claimed that the partner felt a greater need for sex (22%), for 8% they treated sex as an element of life necessary for procreation, 6% of them felt a greater need for sex than a partner, and for 2% sex played an unimportant role in the relationship.

Slightly more than 23% of respondents would expect a gynecologist to talk to them about sexuality during pregnancy, 16% of respondents did not expect it, and the remaining respondents did not think about it (60%). However, during gynecological check-ups, only few women discussed sexuality during pregnancy with their physician in charge (18%). Slightly more than 36% of respondents claimed that their gynecologist raised the topic of sexuality during pregnancy, and when asked about these topics, he freely talked about them. Most of the respondents did not feel embarrassed and had no difficulty talking freely about sexuality with their gynecologist (71%).

Nearly 14% of women said that their gynecologist discussed with her the impact of pregnancy on a woman’s sexuality before or in the early stages of her pregnancy.

While the need to discuss the impact of pregnancy on her sexuality was felt by 13% of the respondents, 53% did not feel the need to discuss it, and the rest did not think about it (35%). Women under 30 years of age felt this need more often (20% of them), older women felt this need less often (6%). This difference was statistically significant (*p* = 0.044).

## Discussion

There are not many data on female sexual functioning during pregnancy.

As it was mentioned in the introduction section, hormonal and physilogical changes during pregnancy alter libido and sexuality in general, which is specific to a particular stage of pregnancy [5]. In our study, majority of respondents felt less need for sex, were less satisfied with intercourses and developed certain SD not experienced before the pregnancy. Due to the enlarged features the preferred sexual position was the “lateral rear to partner” position, and most respondents declared that the lowest sexuality was in the third trimester (66%). Spanish researchers had similar observations to ours—in their study on Quality of Sexuality during Pregnancy, 50.4% of respondents stated that their sexual interest decreased. Aspects like the number of coitus, feeling orgasm with penetration or with masturbation, or the frequency of oral sex, were significantly reduced during pregnancy [18].

Aribi et al. have investigated the impact of pregnancy and the post partum period on the female sexuality and the coupledom and evaluated the quality of information and dialog about this subject [19]. They obverved that sexuality was a taboo for majority of woman. Restraint from intercourse during pregnancy correlated neitherr with education nor with origin. The period of pregnancy and the postpartum have changed the sexual behavior, interfered with the QoL and have disturbed the coupledom [16]. Not all of these observations are consistent with our outcomes. Althought in most cases a decrease in the frequency and quality of sexual intercourse was repoerted in pregnancy, we noted the correlation between sexual activity after getting pregnant and education. The higher the educatio, the more frequent sexual intercourse, which is quite logic, sine better education usually gives better general knowledge and thus less cliches concerning potential harm of sex to pregnancy and fetus. The difference between our and Tunisian findings may be explained by the cultural and religious differences. The attitude toward sexuality in general is more conservative in Muslim countries, which is also indicated by the fact that for majority of Tunisian women sex was a taboo. In our study, although most females felt the need to talk about the impact of pregnancy on their sexual life, only few of them discussed the issue with the gynecologist, which shows that sex is still some kind of awkward topic in Poland. Alike the Tunisian team we also reported that pregnancy had rather negative impact on sexualioty in terms of frequency and quality of intercourse. However, in contrary to them, Polish women have not observed the negative influence of their pregnany on the coupledom – both the relationship and the self and partner’s perception of the changed body were positive.

The Thai team has determined the changes of sexuality during pregnancy and explored women’s attitudes and sources of information concerning sexuality during pregnancy [20]. The authors observed that sexuality decreased significantly throughout pregnancy in those women. The majority of them were concerned about the adverse effects of sexual intercourse on pregnancy. These outcomes are consistent with our findings–we also reported decreased sexuality during pregnancy and lowest needs were declared in the third tremester. The results also indicate the need for counseling–the greater the knowledge, the less fears concerning sex during pregnancy.

In the Thai study, the main concern was the potential harm to the fetus. And 62% of pregnant women received information about sexuality during pregnancy from their health physicians. Polish women also declared fear of the potential risk to the fetus as the main reason to restrain from. sex during pregnancy, however, in only 14% of cases the gynecologist raised that issue. It may be explained by the fact that patients still find sexuality a taboo, an awkward topic to discuss and/or that gynecologists are not aware enough of the imprtance of this problem.

In another Thai study a potential sexual dysfunction in pregnant women was rather high. Sexuality was decreased significantly throughout pregnancy. However, most of the pregnant women were not concerned about decreasing sexual desire during pregnancy [21].

These observations are consistant with ours, sincee Polish women in our study also experienced SD more frequently after getting pregnant than before it, and the lowest sexual needs were during the thitd tremester.

Blumenstock et al. in their study to assess changes in the frequency of sexual intercourse across all weeks of pregnancy, confirmed that sexual intercourse frequency clearly declined across pregnancy, yet the pattern followed the course of common pregnancy symptomology (i.e., nausea, fatigue) more closely than trimester cutoffs [22]. This observation shows that the changes in sexual frequency are more complex than the general declines suggested by other studies.

Serati et al. have reviewed the available evidence to define present knowledge about female sexual function during pregnancy and after childbirth [23]. In their metha-analysis sexual function was found to have a significant global decline during pregnancy, particularly in the third trimester. The lack of adequate information about sex in pregnancy and concerns about the possible adverse obstetric outcomes were the most relevant factors responsible for the avoidance of sexual activity during pregnancy [18]. Our outcomes are consistent with the literature and they emphasize the need for information, education and counseling about sexuality during pregnancy.

Self-esteem is very important for female’s sexuality. In our study, majority of the respondents’ partners assessed the attractiveness of the respondent in the same way as before the pregnancy (77%), and most of the respondents did not consider that after becoming pregnant they would be less attractive to their partner and that pregnancy would adversely affect their relationships and sexual life (35%). Similar results were presented in the Turkish study—6 out of 10 pregnant women had a positive attitude toward sexuality during pregnancy, and their sexual self-efficacy (26.35 ± 6.71) and sexual self-consciousness (24.75 ± 9.10) levels were moderate [24].

The advantage of our study is a pretty large homogenous group of women. However, the disadvantage is the fact that the survey was performed in the private clinic on physiologically pregnant women, therefore it may not be representative of the entire population of Polish pregnant women.

## Conclusion

Pregnancy has significant impact on woman’s sexuality. After becoming pregnant majority of women experience less need for sexual intercourses, decreased number of intercourses with less satisfaction, that progress significantly throughout pregnancy. The fear of the potential risk to the fetus as the main reason to restrain from. Sex during pregnancy indicates the great need for gynecological counseling.
